# Double BioDisk: a new bioprosthetic device for transcatheter closure of atrial septal defects - a feasibility study in adult sheep

**DOI:** 10.2478/v10019-012-0029-8

**Published:** 2012-05-30

**Authors:** Dusan Pavcnik, Kurt Tekulve, Barry T. Uchida, Zhong-Huo Luo, Miran Jeromel, William G. Van Alstine, Frederick S. Keller, Josef Rösch

**Affiliations:** 1 Dotter Interventional Institute, Oregon Health Sciences University, Portland, Oregon, USA; 2 Cook Medical Incorporated, Bloomington, Indiana, USA; 3 School of Veterinary Medicine, Purdue University, West Lafayette, Indiana, USA; 4 Institut of Radiology, University Clinical Center, Ljubljana, Slovenia

**Keywords:** atrial septal defect, transcatheter closure, small intestinal submucosa, biomaterial, embolism, heart septal defects

## Abstract

**Background:**

To evaluate the long-term effectiveness and safety of a new Double BioDisk (DBD) device for closure of atrial septal defect (ASD).

**Materials and methods.:**

ASD was created with transeptal needle (TS) followed by balloon dilatation in 12 sheep weighing 40.1 to 64 kg (mean 55.2 ± 7.1). The ASD diameters were measured after creation and two weeks later before DBD implantation. The DBDs consists of two nitinol rings 18 to 28 mm in diameter connected with small cannulas and covered with a porcine small intestinal submucosa (SIS). They were implanted via a 10 Fr sheath. DBD effectiveness was evaluated by angiocardiography and by intra-cardiac echogram (ICE) with Doppler studies. Two animals were acute, two were followed for 6 weeks, three for 3 months, three for 6 months and two for 12 months.

**Results:**

TS punctures were successful in 10 sheep. In two sheep ASD was created by existing PFO dilation. The ASD size ranged from 13–15 mm (mean 14.1± 0.73 mm) after initial balloon dilation and from 9–13 mm (mean 10.06 ± 1.37 mm) after two weeks. In all animals none of the successfully implanted DBDs spontaneously embolized on release or on follow up. ICE demonstrated no shunting around the DBDs during follows ups. Macroscopic and histologic evaluation of the 6, 12, 24 and 52 weeks animals showed that DBDs were well incorporated in the atrial septum with complete shunt closure. The SIS showed progressive remodeling with the host cells, including endothelization of the DBD devices.

**Conclusions:**

ASD closure with the Double BioDisk is safe and effective in adult sheep.

## Introduction

Since King and Mills reported percutaneous treatment of an atrial septal defect (ASD) in the 1970s, many transcatheter ASD closure devices have been developed.^1^–^5^ These ASD closure devices have been also used for patent foramen ovale (PFO) closure. We developed and tested a single disk device covered with porcine small intestinal submucosa (SIS) – BioDisk (BD) - for the closure of PFO in a piglet model.^6^ For closure of ASD in large animals we developed a Double BioDisk (DBD) covered with SIS. We report on the feasibility, long-term effectiveness and safety of DBD application in adult sheep with percutaneously created ASD. Sheep were used for testing, since the DBD biological cover is of a porcine origin.

## Material and methods

The study protocol was approved by the institutional Animal Care and Use Committee. Twelve adult weighing 40.1 to 64 kg (mean 55.2 ± 7.1 kg) were used for testing the DBD device. A cardiac mobile system (GE/OEC 9800; GE Medical Systems, OEC, Salt Lake City, UT) with digital imaging was used for fluoroscopy and angiocardiography. Angiocardiography was performed with an injector (Medrad Mark Plus, Medrad, Inc. Warrendale, PA). For intracardiac echocardiographic (ICE) studies, the AcuNav System (Acuson, Siemens Inc., Mountain View, California) was used.

### Percutaneous transcatheter creation of ASD

Preparation of animals and their anesthesia were described in previous paper.^7^ Electro cardiogram (EKG), heart rate, oxygen saturation and end tidal CO_2_ were monitored during the procedure. After induction of general endotracheal anesthesia, the sheep were secured with their back on the radiographic table with their hind limbs in moderate abduction. The neck and the right groin were shaved and prepped. 6 F vascular sheath was percutaneously placed into the right jugular vein and a short 14 F sheath into the right femoral vein. The jugular vein sheath was used for physiologic monitoring, the femoral vein sheath for procedure performance.

The ASDs were created percutaneously with modified transeptal needles (TS) followed by 14 mm balloon dilatation (Cook Medical, Bloomington, IN) as previously described.^8^ The stretched ASD diameter was then measured with a Coda balloon catheter (Cook Medical). The animals were then recovered and returned to the Department of Comparative Medicine (DMC) for monitoring by veterinarians. Fourteen days after ASDs creation, the sheep were restudied by angiocardiography, ICE study and Coda balloon measurement of the shunts. DBDs were then implanted.

### Double BioDisk (DBD) device

**FIGURE 1:**
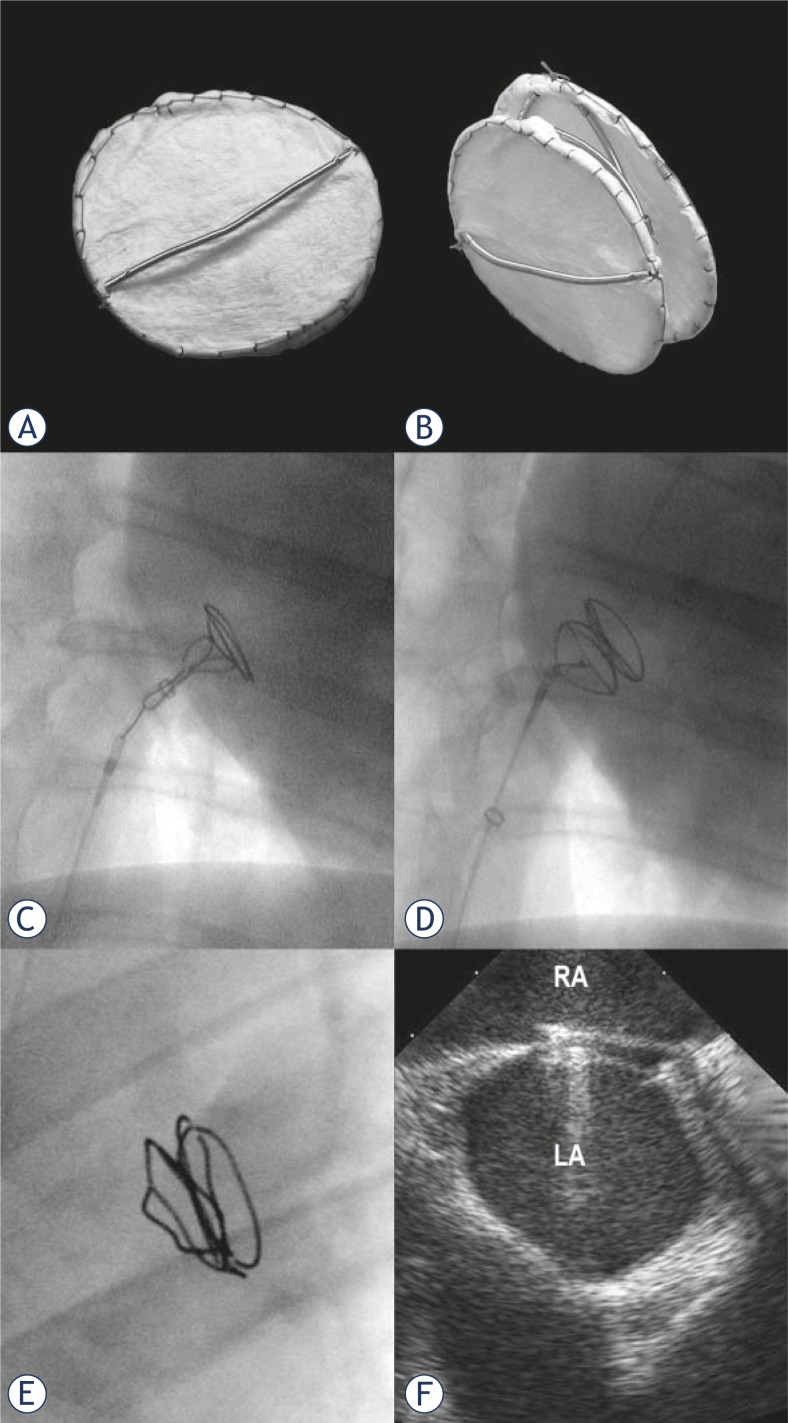
The double BioDisk (DBD) ASD occlusive device 18 mm in diameter. A. Right atrial side with delivery bar. B. Oblique projection. C. Deployment of the left atrial DBD disk. D. Deployment of DBD across ASD with delivery bar still attached to the delivery wire. E. X-ray of DBD at 3 months. F. Intracardiac echocardiogram 6 months after DBD deployment shows device with thickened discs. No shunting was seen.

The DBD were constructed as a joint effort between Cook Medical and the Dotter Institute specifications. The DBD consisted of two nitinol rings covered with platinum coil. Both flexible rings were connected with small cannulas and covered with SIS. The cross bar of the right atrial disk was the delivery bar. SIS was sutured with Prolene 6.0 to the radio-opaque rings (Figure 1AB). The DBD was lyophilized and then preloaded by the manufacturer into a 10 Fr cartridge. The delivery system was similar to the system used in the jugular Tulip filter delivery system (Cook Medical). The DBD is self-expanding and self-centering device. The DBD sizes for this study were 18 mm, 23 mm and 28 mm in diameter. Device to defect ratio of 1.8 or larger was used.

### DBD closures of ASD

Preparation of animals, their anesthesia and sheaths placements were similar as for ASD creation. The animals received heparin in dose of 100 IU/kg of body weight. Intravenous saline was administered as needed and respiration rate, expired carbon dioxide, oxygen saturation and EKG were monitored during the procedure. A 5 Fr multipurpose catheter (Cook Medical) was introduced through the femoral vein sheath into the right atrium to catheterize the ASD. A 0.035” Road Runner guide wire (Cook Medical) was used to advance the catheter under fluoroscopy into the left atrium. The Road Runner was then replaced with a long, 0.035” stiff Amplatz wire (Cook Medical). To measure diameter of the ASD, a Coda balloon (Cook Medical) was used. An hourglass appearance of the Coda balloon was indicative of ASD size. No attempts were made to dilate the ASD. After the Coda balloon was removed, a10F Flexor sheath (Cook Medical) was advanced across the ASD into the left atrium.

The preloaded DBD was first rehydrated with injection of 10–20 ml of heparinized saline into the cartridge. The delivery bar of the right atrial disk of the DBD was then connected to a hook of a stiff introducing wire. The DBD was introduced into the Flexor delivery sheath and advanced through the ASD into the left atrium. The left atrial disk was then delivered by holding the DBD and by withdrawing the delivery sheath (Figure 1CD). Since the disk is self expanding, it assumed its original rounded shape in the left atrium after exiting the sheath. The DBD was then pulled back against the septum while the other disk connected to the delivery bar was still inside the Flexor sheath in the right atrium. Before releasing the device, the sheath was pulled back to expose the right atrial disk with delivery bar still connected to the hook. Prior to deployment, the DBD position was assessed by contrast injection into the right atrium in the RAO view. When no reflux into the left atrium was found, delivery bar of the DBD was released from the holding catheter. The DBD sizes used for ASD closure were 18 mm in 5 sheep, 23 mm in 4 sheep and 28 mm in one sheep.

After DBD deployments angiocardiograms in left anterior oblique and lateral views followed to document the position of the DBD and its effectiveness in occluding the ASD. The delivery catheter and 10 Fr sheath were then removed and replaced with an ICE catheter to evaluate DBD effectiveness with Doppler studies. Two animals were acute; two were followed for six weeks, three for 3 months, three for 6 months and two for 12 months.

### Acute studies

In two acute animals, DBD repositioning and removal were tested prior to delivery catheter removal. First the DBD was expanded and then retracted into the delivery sheath. Then the entire DBD was deployed, but not released. Afterwards it was pulled back into the sheath and redeployed into the ASD. To test its retrievability, the DBD was removed from its ASD position and intentionally embolized into the right atrium. Both the Amplatz gooseneck snare and vascular forceps were used for the retrieval of the embolized DBDs. After retrieval a new DBD was deployed and after 3 hours observation, the acute animals were euthanized. The harvested hearts were cut longitudinally and photographs of the atrial septums were made.

### Follow-up studies

Ten chronic animals were recovered after the procedure and returned to the DMC where veterinarians checked them on a daily basis.

Follow-up angiocardiograms of the right atrium and ICE with Doppler studies were done at 6 weeks (n=2), 3 months (n=3), 6 months (n=3) and 12 months (n=2) after DBD placement under the same procedure protocol as in initial studies. After satisfactory views of the DBD effectiveness for ASD closure were obtained, the animals were euthanized and their hearts harvested. After photographs of the atrial septa, the hearts was preserved in buffered formalin and were sent for detailed histological evaluation. Each DBD specimen was embedded in methylmethacrylate blocks. Four cross sections of each device were processed using plastic embedding. All slides were stained with hematoxylin and eosin (H and E) stain using routine methods.

## Results

The ASDs were created safely in all 12 sheep and did not lead to any complications. In 10 sheep ASD were created by puncture and balloon dilation at the fossa ovalis, in the other 2 sheep by balloon dilation of the existing PFO. The stretched ASD size immediately after balloon dilation ranged from 13 to 15 mm (mean 14.1 ± 0.73 mm). At 2 weeks prior to DBD placement the ASD size ranged from 9–13 mm with a mean 10.06 ± 1.37 mm (Table 1).

All DBDs were successfully implanted and none spontaneously embolized on release or on follow up. In the first two acute animals, the DBDs were easily retracted into the delivery sheath after their partial or full deployment and redeployed into the ASD. The two intentionally embolized DBDs into the right atrium were safely retrieved and new DBDs were placed into the ASD. Angiocardiograms after DBD deployment showed good placement and absence of shunting. ICE evaluation with Doppler studies did not reveal any shunting around the DBDs. EKGs did not demonstrate any arrhythmias. In the chronic studies, the DBDs were highly visible on x-ray (Figure 1E). There were no fractures of the device frame. Follow up angiocardiograms and ICE Doppler studies documented complete closures of the ASDs and no shunting in all 8 sheep (Figure 1F).

### Gross examination

**FIGURE 2:**
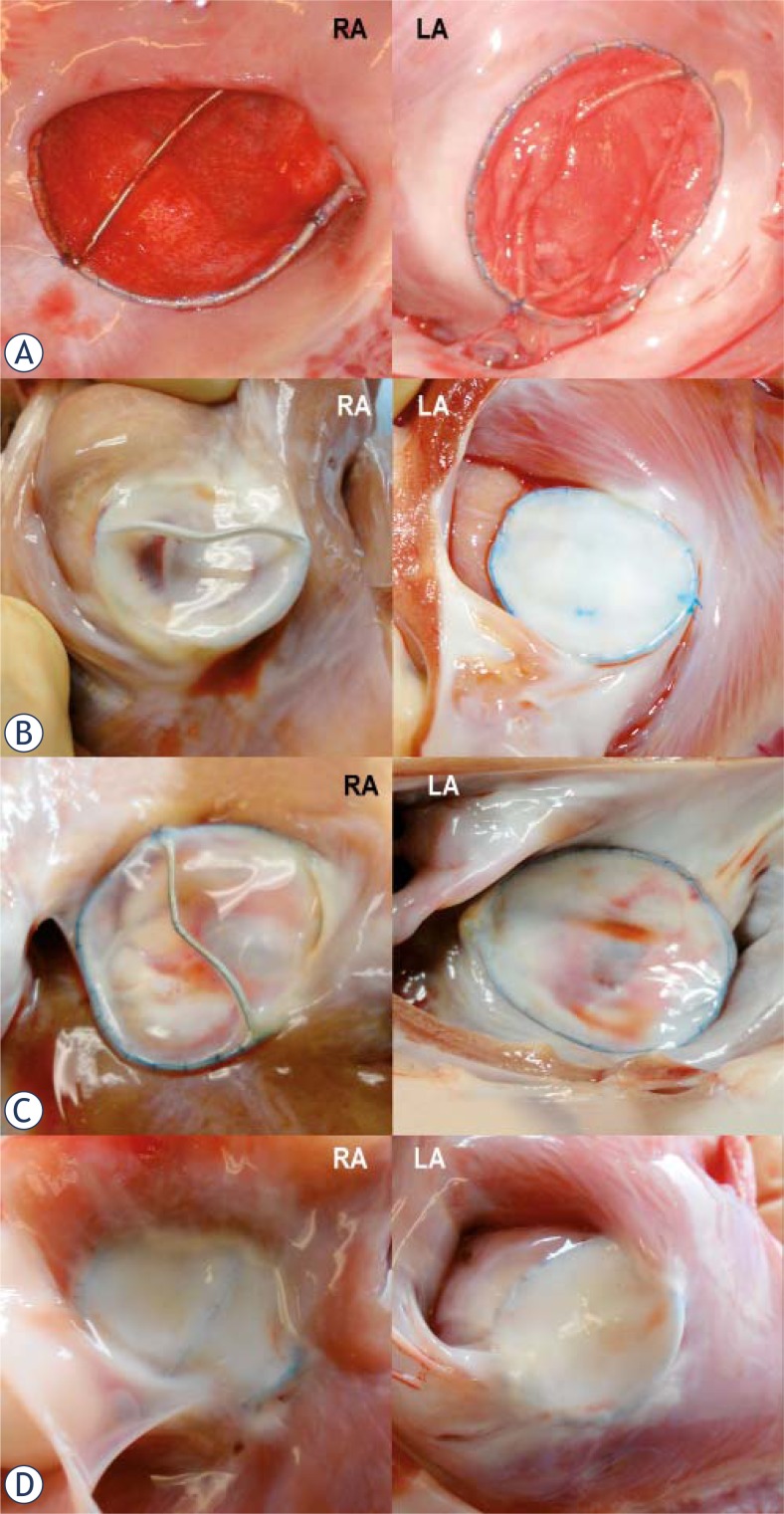
Gross specimens of the deployed double Biodisks into the adult sheep ASDs. A. Three hours after deployment, thin layers of early thrombus cover right and left atrium disk of the 28 mm device. B. At 3 months, the 18 mm DBD is almost completely incorporated into myocardium. Glistening disk surfaces indicate complete endothelization. C. At 6 months, the DBD is almost completely incorporated into myocardium of the right and left atrium. D. At 12 months, the DBD is completely incorporated into myocardium of the right and left atrium.

The implanted DBDs in all animals, acute and chronic, were well self-centered and attached to the ASDs with good apposition to the interatrial septum and adjacent myocardium (Figure 2). Both discs were flattened against the intra-atrial septum and held in place by the connection between the DBD. The DBDs did not obstruct blood flow in to coronary sinus or pulmonary veins nor did it compromise mitral or tricuspid valves. In two acute animals euthanized 3 hours after DBD placement both discs were covered by thin red layer of early thrombus (Figure 2A). In two animals euthanized at 6 weeks, the SIS surfaces on both disks were shiny, transparent and without thrombus. Disks were partially incorporated into the myocardium. In three animals sacrificed at 12 weeks, DBD rings were almost completely incorporated into the myocardium wall. The SIS of the left and right atrium disks were well apposed to the myocardium excluding the ASD opening. The SIS was glistering and shiny indicating endothelization (Figure 2B). The SIS was less transparent than that at 6 weeks. At 24 weeks and 52 weeks DBDs were free from adjacent important cardiac structures in all of the specimens. The SIS membranes of the DBDs were positioned with a good apposition across the ASD and fused together with myocardium (Figure 2C). At 12 months, the disks apposition and incorporation into adjacent myocardium was complete, in the right and in the left atrium (Figure 2D). Glistening SIS surfaces were seen in all specimens. The delivery crossbars of the DBDs were covered with shiny tissue and partially fused with device.

### Histological evaluation

**FIGURE 3:**
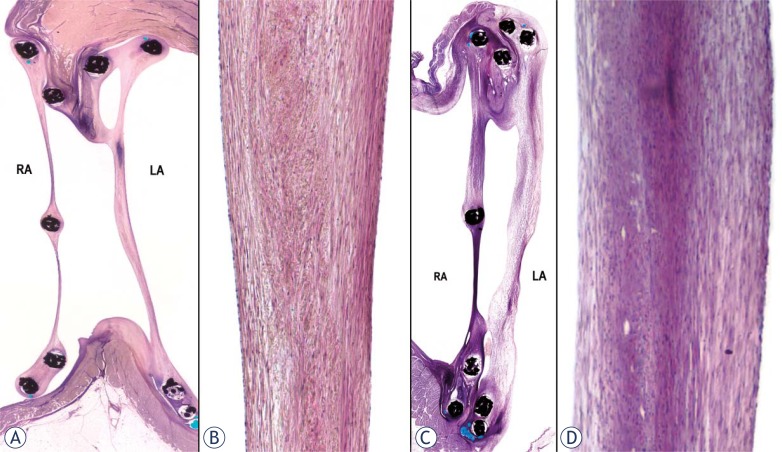
Histologic cross sections of the deployed double BioDisks into the adult sheep ASDs. A. At 3 months, the right atrial disk of the 18 mm device is apposed and the left atrial disk is incorporated into myocardium. The crossbar is incorporated into the right disk. The disk thickness ranges between 0.1 to 0.25 mm at the right atrial disk and between 1 to 2 mm at the left atrial disk. (Low magnification Hand E) B. At 3 months the center of the left disc is partially remodeled into neointima/connective tissue and contains no inflammatory cells. Remnant SIS is visible in the core of the leaflet. (H and E stain, 40x) C. At 6 months, the 18 mm disks are completely imbedded into myocardium and the distance between them ranges from 2 to 3 mm. The crossbar is incorporated into the right disk. Right atrial disk thickness ranges from 0.4 to 0.8 mm and left atrial disk thickness from 1.5 to 3 mm. (Low magnification Hand E) D. At 6 months the center of the right atrial disk is remodeled into mature neointima/connective tissue and contains no inflammatory cells. Remnant SIS is not visible. All luminal surfaces are lined by endothelium (H and E stain, 40x).

Histological sections through the center of the DBDs exhibited well covered ASDs by the device disks. At follow up, there was progressive device apposition and incorporation of disks with adjacent myocardium, progressive SIS remodeling into neointima-connective tissue and progressive SIS endothelization. At six weeks follow up, almost 85% of the rings on the right and 50% on the left atrial side were incorporated into the myocardium and surrounded by fibrous tissue less than 200 microns thick. The residual ring parts not embedded in the myocardium were surrounded by fibrous connective tissue and lined by endothelium. The SIS thickness ranged from 0.1 mm in the center to 2.5 mm near the rings and was covered by spindle shaped cells forming a neointima. Flat endothelium covered most of the SIS. The crossbars were surrounded by fibrous tissue. Only minimal inflammatory changes with a few lymphocytes were seen. At 3 months, approximately 95% of the rings on the right side and 60% on the left side were embedded into adjacent myocardium and surrounded by fibrous tissue less than 0.1 mm thick. The SIS showed progressive remodeling into fibrous connective tissue. The SIS disks were 0.1–0.5 mm thick at their centers and 1.5–3 mm at their periphery. The right disk was thinner than the left disk (Figure 3AB). All discs surfaces were covered by mature endothelium. Crossbars were surrounded by neointima and incorporated into disks. At 6 months, the disks apposition and incorporation into adjacent myocardium was almost complete, 96% in the right atrium and 85% in the left atrium (Figure 3C). The ring wires not embedded into the myocardium were surrounded by neointima lined by endothelium. The SIS discs were composed of mature neointima consisting of fibrous connective tissue. SIS collagen fibers were not observed indicating complete remodeling (Figure 3D). The disks were 0.4–0.9 mm thick at the center and 1.5–3mm at the periphery. The crossbars were incorporated into the disk (Figure 3C). All lumen surfaces of the discs and crossbars were covered by flat mature endothelium.

## Discussion

Adult sheep were used to test the DBD for several reasons. We reported several minimally invasive studies in sheep and we are familiar with this model.^9^,^10^ The size of their heart size approximates the size of the human heart and allows large closure devices to be tested. Adult sheep have a stable body size and when compared to swine that continue to grow and put on weight.^6^,^11^ They allow for long-term follow-up. In addition, the sheep coagulation and fibrinolytic systems are closer to that of humans compared to those in canine or swine.^12^ Since the DBD cover is composed of swine origin, SIS is a xenogenic biomaterial to sheep.^13^ The cardiac anatomy of sheep with a more anteriorly positioned heart has a significantly steeper interatrial septum compared to humans that can be challenging for TS puncture. A long TS needle was developed corresponding to the length of sheep body with a curved needle tip allowing TS punctures to be easily and safely accomplished in all of our animals.^8^ We always punctured and established the ASDs in the area of the fossa ovalis because it allows for a mature stable ASD size. Two weeks after its creation, the ASD size in our animals decreased slightly from a mean 14.1 ± 0.73 mm to a mean 10.06 ± 1.37 mm. The ASD creation was not attempted in the septum secundum because in sheep this structure is significantly thicker than it is in humans. Previous experience showed us that punctures followed by dilations at the septum secundum do not lead to stable ASDs. They heal in two weeks.^8^

This is our third generation device for cardiac septal closure. The first one, the Monodisk, was developed in the early nineties.^4^ It consisted of a stainless steel ring covered with two layers of nylon mesh that was positioned on the left atrial ASD side. Three coiled stainless steel wires on the back side of the Monodisk served for its delivery and anchoring at the right atrial ASD side. A 9F introducing sheath was used for its delivery. Six months follow-up studies in canines showed it was effective for closure of ASD 8–10 mm in size. However problems with the Monodisk were its rigid frame, complex delivery, difficult post delivery retrieval and our inability to find a FDA approved nylon mesh alternative.

The second generation of the disk closure device – the Biodisk – was developed about 15 years later after we gained experience with nitinol and SIS. The nitinol that was used as a pliable material for the disk frame and the biomaterial SIS was used for the covering. The Biodisk was intended for closure of PFO. It consisted of one disk inserted into the left atrial ASD side. Nitinol wires of the disk were covered by platinum coil and a crossbar attached to the disk was used for delivery and for anchoring on the right atrial ASD side. An 8F sheath was used for Biodisk delivery. Initial testing in piglets showed its easy delivery, good retrievability and long-term effectiveness for closure of PFO measured with the 10–14 mm balloons.^6^

The DBD with two disks was developed for closure of ASD. The Biodisk with only one disk would not be sufficient for the occasional complex anatomy and large size of ASDs. The DBD feasibility study demonstrated that this new bioprosthetic device has excellent potential for ASD closures. The DBD was easy to deploy, it self-centered during deployment and covered the entire ASD without encroachment on structures in both atria. When needed, the DBD could be repositioned or retrieved if released inappropriately or lost as shown after its intentional embolization. The DBD showed excellent effectiveness for ASD closures and no residual shunting was seen at ICE examinations with color Doppler studies. The gross studies showed good apposition of the disks to the septum and myocardium without compromise of valves and other cardiac structures. At 3 hours after DBD placement, a thin layer of early thrombus covered the SIS disks as initial the phase of neointimal formation. Otherwise, no thrombus was found on the follow-up studies as the DBDs rapidly endotheliolized. The histologic follow-up studies then revealed progressive apposition leading to full incorporation of the DBD disks with adjacent myocardium, progressive SIS remodeling into connective tissue and complete DBD endothelization. These healing processes were well demonstrated on the histologic DBD cross sections. The space between the disks, originally separated by thick septum secundum and residual septum primum progressively decreased by neointimal formation and some atrophy of residual septum. The neointimal formation started at the disks periphery, extended to the center of the disks. It was more pronounced on the left atrial SIS disks. The 6 and 12 months follow-up showed complete SIS remodeling into the heart connective tissue.

The DBD feasibility study did not compare the biomaterial SIS with septal occluders covered with synthetic fabrics. This was done in detail by Jux *et al.* in 2006.^14^ They compared the first septal occluder device with biodegradable matrix, the Biostar covered with the purified intestinal collagen layer (ICL) with the Starflex covered with a knitted polyester fabric. The ICL, similar to SIS, originated from porcine small intestinal mucosa and both had similar thickness between 150–200 microns. A 10F sheath was used for deployment of both the Biostar and DBD. The study by Jux *et al.* in young sheep that from 7 days to 2 years showed distinct advantages of the biomaterial matrix. Biostar had decreased thrombogenicity, particularly when the device was heparin coated. It showed accelerated healing with early endothelization and low immune response with fast ICL remodeling into connective cardiac tissue. Because of these positive results, the Biostar has already been applied successfully in treatment of ASD in children and adults.^15^,^16^,^17^

## Conclusions

Long term both right and left atrial SIS disks were remodeled into the heart connective tissue, so that only a minimal amount of metal spring material has been left behind. ASD closure with the Double BioDisk is safe and effective in adult sheep.

## Figures and Tables

**TABLE 1:** Results of percutaneous atrial septal defect closure

**Animal No**	**Weight kg**	**Stretched ASD diameter mm**		**DBD size mm**	**Placement successful Yes or No**	**Follow Up Weeks**	**ICE Follow Up**
		**day 1**	**day 14**				
1	49.8	15	acute	28	Y	acute	no shunt
2	57.6	13	acute	23	Y	acute	no shunt
3	60.7	14	11	23	Y	6	no shunt
4	61.6	14	10	23	Y	6	no shunt
5	44.3	15	9	18	Y	12	no shunt
6	47.4	14	9.5	18	Y	12	no shunt
7	53.2	14	9	18	Y	12	no shunt
8	46.3	13	10	18	Y	24	no shunt
9	64.0	15	13	23	Y	24	no shunt
10	46.2	14	9	18	Y	24	no shunt
11	40.1	14	9.2	18	Y	52	no shunt
12	52.2	15	9	18	Y	52	no shunt

ASD=atrial septal defect, DBD=Double BioDisk, Y=yes, ICE= Intra-cardiac Echogram
